# Surgical reconstruction of traumatic ciliary body dialysis: a case report

**DOI:** 10.1186/s13256-016-1170-6

**Published:** 2017-01-23

**Authors:** Adam Kluś, Mariusz Kosatka, Milena Kozera, Marek Rękas

**Affiliations:** Department of Ophthalmology, Military Institute of Medicine, 128 Szaserów St., 04-141 Warsaw, Poland

**Keywords:** Trauma, Cyclodialysis cleft, Persistent hypotony, Lens subluxation

## Abstract

**Background:**

A cyclodialysis cleft is a gap resulting from disruption of the longitudinal fibers constituting the ciliary body attachment to the scleral spur. The cyclodialysis cleft can be of traumatic or iatrogenic origin, and it may occur during intraocular surgery or as a result of a glaucoma operation. In this report we present a surgical technique to treat cyclodialysis: cyclopexy combined with phacoemulsification subluxation lens, transscleral suturing of Cionni ring, and intraocular lens implantation with iris cerclage suture.

**Case presentation:**

A 44-year-old Polish woman experienced a traumatic cyclodialysis cleft in her left eye, complicated by persistent hypotony, maculopathy, lens subluxation, and pupillary sphincter injury. Her corrected distance visual acuity was 0.1 (Snellen chart) and intraocular pressure 3.0 mmHg. We performed direct cyclopexy, anterior vitrectomy, removal of the subluxated lens by phacoemulsification, followed by an insertion of a capsular tension ring with 1-point scleral suture fixation with implantation of intraocular lens in the capsular bag and suturing around the pupil. Anterior segment optical coherence tomography revealed closure of the cleft by reattachment of the ciliary body to the sclera spur. Her corrected distance visual acuity was 0.8 and intraocular pressure 18 mmHg.

**Conclusions:**

The choice of operating technique depends on the area of the ciliary body dialysis, the number of clefts and their location, the presence of other abnormalities of the ocular structures, and the surgical skills of the operator. Cyclopexy combined with phacoemulsification and transscleral suturing of Cionni ring and intraocular lens implantation with iris cerclage suture can be a good solution in cases of this type. The applied surgical technique proved to be effective.

## Background

A cyclodialysis cleft is a gap resulting from disruption of the longitudinal fibers constituting the ciliary body attachment to the scleral spur [1]. The cyclodialysis cleft can be of traumatic or iatrogenic origin; it may occur during intraocular surgery, for example extracapsular cataract extraction, phacoemulsification, secondary intraocular lens (IOL) placement, or phakic IOL removal or as a result of a glaucoma operation (trabeculectomy, trabeculotomy, goniotomy) [2]. Clinical symptoms such as hypotony, shallow anterior chamber (AC), abnormal anterior segment architecture, cataract, retinal and choroidal folds, hypotonous maculopathy, and loss of vision in cases of prolonged hypotony, can cause the ophthalmologist to suspect cyclodialysis cleft, and a detailed ophthalmological examination based on ultrasound biomicroscopy (UBM) or anterior segment optical coherence tomography (OCT; Visante OCT™) should be performed [1–5]. Here we describe a treatment method based on the use of phacoemulsification and a capsular tension ring (CTR) for *ab externo* and *ab interno* cyclodialysis repair and reconstruction of the pupillary sphincter.

## Case presentation

**Fig. 1:**
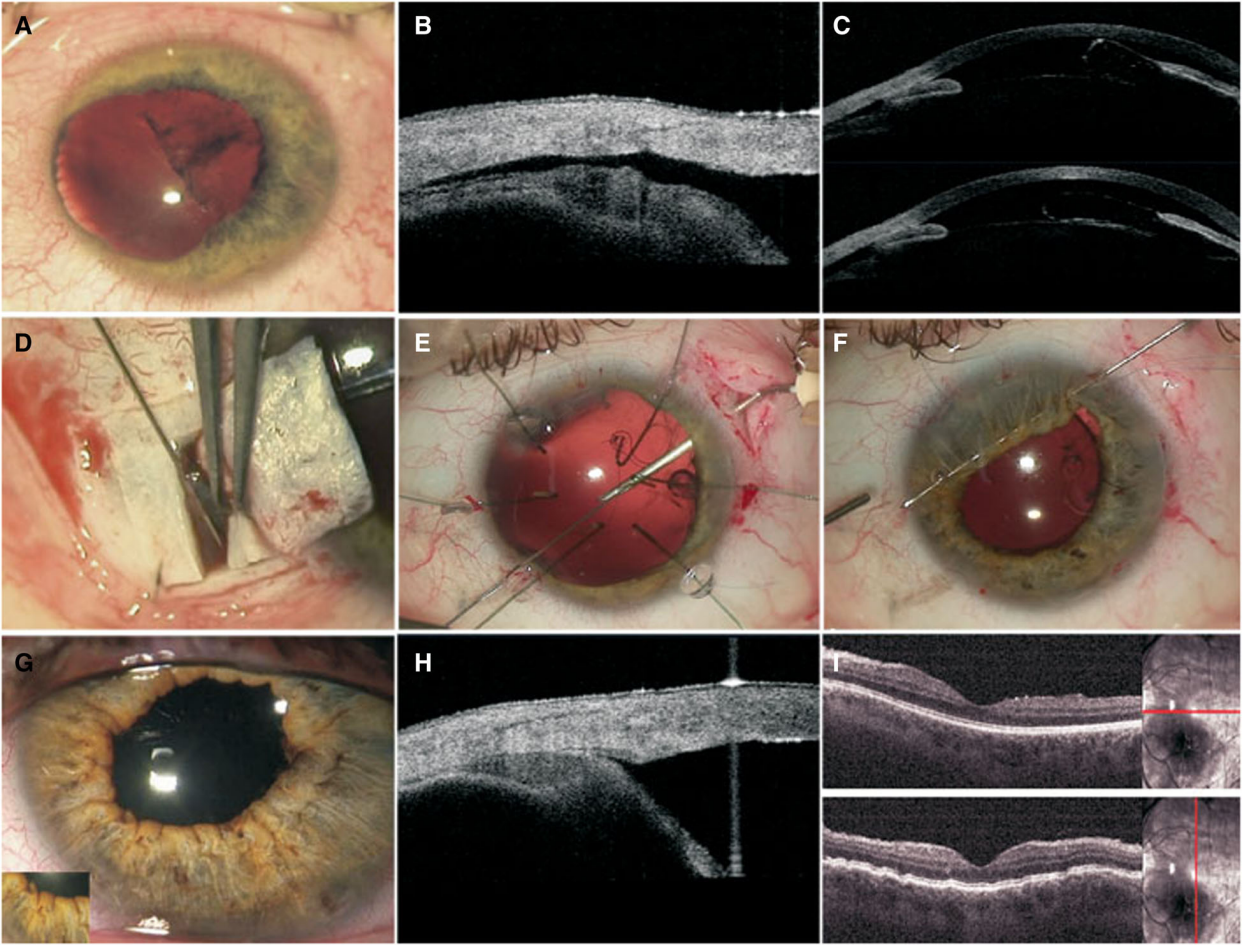
**a** Iris sphincter tear, mydriatic pupil, subluxated lens with iridophacodonesis, **b** OCT-Visante visualized cyclodialysis cleft and choroidal detachment, **c** OCT-Visante visualized shallow AC, subluxated lens, vitreous body prolapse into the anterior chamber, **d** Intrasurgery photo: scleral tunnel in the temporal quadrant and point limbic opening and fix the ciliary body to the deep scleral flap, **e** Intrasurgery photo: the retractors that were fixed to the anterior lens capsule to ensure its stability, **f** Intrasurgery photo: Iris suture. **g** Slit lamp examination - anterior segment 6 months after the surgery, **h** Visante OCT 6 months after the surgery, **i** Macular OCT 6 months after the surgery

A 44-year-old Polish woman who was involved in a car crash presented to our Military Institute of Medicine with a head injury and blunt ocular trauma: she had trauma to her left eye with eye pain and decreased visual acuity. The best-corrected visual acuity (BCVA) of her left eye was 0.1 according to Snellen and the intraocular pressure (IOP) was less than 3 mmHg. A slit lamp examination showed corneal edema with Descemet membrane folds, shallow AC, iris sphincter tear from 7 o’clock to 11 o’clock position, mydriatic pupil, subluxated lens from 1 o’clock to 5 o’clock with iridophacodonesis, and vitreous body prolapse into the AC (Fig. 1a). A gonioscopy and anterior segment OCT (Visante OCT™) confirmed cyclodialysis cleft and choroidal detachment in the nasal and superior temporal quadrant (Fig. 1b, c).

A fundus examination also showed star folds and macular edema with mean macular thickness of 745 μm, and 396 μm in the central part of her fovea. She was treated with Tropicamidum (tropicamide) 1% and topical steroids. No improvements in her visual acuity, IOP or anatomy were observed after 2, 4 and 6 weeks.

### Surgical technique

Surgery was performed under retrobulbar anesthesia. Retrobulbar block is a useful method for intraocular and orbital surgery. First, the bulbar conjunctiva was detached from the limbus within the nasal quadrant. Then a scleral incision was performed and a partial thickness sclera flap 3-mm wide and 2-mm long was dissected. Point entries into AC were made at 3 o’clock and 11 o’clock positions. Sodium hyaluronate (1%) was applied into the AC to reform and stabilize the surgical planes and protect the endothelium. The next step was the scleral bed incision and dissection of a deep rectangular scleral flap 2×1 mm in size with ciliary body exposure. The iris was pulled back from the prepared fistula. Subsequently, two 10.0 nylon sutures were used to fix the ciliary body to the deep scleral flap. Then interrupted 10.0 nylon sutures were used on both scleral flaps and single interrupted conjunctival sutures were applied. Topical dexamethasone (0.1%), Levofloxacinum (levofloxacin), and diclofenac were applied postoperatively for a month. In this period her left eye BCVA was 0.1 to 0.5 according to Snellen, and IOP was 4 to 6 mmHg. Gonioscopy and Visante OCT™ performed 1 week after the surgery showed that the ciliary body had reattached to the scleral spur, closing the cleft but only at the sutures. However, over and under the sutures and in the superior temporal quadrant, cyclodialysis cleft and choroidal detachment were detected (Fig. 1c). A fundus examination confirmed the presence of star folds and macular edema.

Considering the above, our patient qualified for a revision surgery. In the first step retrobulbar anesthesia was applied and 2.6-mm self-sealing corneal tunnel in the temporal quadrant and point limbic opening at 11 o'clock were performed (Fig. 1d). Sodium hyaluronate (1% and 4%) was applied into the AC to reform and stabilize the surgical planes and protect the endothelium. A continuous curvilinear capsulorhexis (CCC) was made and then additional limbic openings at 1, 5, and 7 o'clock were used to insert the retractors that were fixed to the anterior lens capsule to ensure its stability (Fig. 1e). Following that, an anterior vitrectomy was performed. The nucleus was removed using the phaco chop phacoemulsification technique. The limbus was also incised at 9 o'clock and 2 o'clock, and other retractors supporting the AC were introduced. Cortical masses were removed. Next, the conjunctiva and the superficial scleral flap in the superior temporal quadrant were dissected. A 13-mm Morcher MR-1 L Cionni-modified CTR was then inserted into the capsular bag. The CTR was sutured with 10.0 nylon to the sclera at 1 o'clock, 1 mm posterior to the surgical limbus, and at 1 o’clock through the ring eyelet to the site of the cyclodialysis. A +23.5 diopter, foldable IOL (MA60BM) was implanted into the capsular bag through a 2.6-mm corneal tunnel. Then 10.0 nylon suture was placed through the iris and around the pupillary sphincter with its simultaneous centering. Following dissection of the conjunctiva in the nasal quadrant, a parallel 3-mm sclerotomy was made approximately 1 mm from the limbus. Two opposite sutures were placed *ab interno* at the base of the iris, using a straight needle with Prolene 10-0, through a 3 o'clock incision position on the corneal limbus (Fig. 1f). Both stitch ends were tied and hidden inside the sclerotomy. Corneal incisions were inspected for leaks and interrupted sutures were placed on the scleral and conjunctival flaps. Postoperative medications included topical 0.1% dexamethasone, 0.5% Levofloxacinum (levofloxacin; Oftaquix), and diclofenac (Naclof), four times a day for a month.

For 5 months after the surgery her BCVA was 0.4 to 0.8 according to Snellen, and IOP was 10 to 22 mmHg. Six months after the procedure the values stabilized at 0.7 for BCVA and on average 14 mmHg for IOP. A physical examination revealed no deviations within the anterior segment, her pupil was normal in size and equal, and IOL was in a correct central position (Fig. 1g). Visante OCT™ showed cleft closure and complete resolution of the choroidal detachment (Fig. 1h). A stereoscopic fundus examination and OCT confirmed no signs of edema, and the average macular thickness was 304 μm and 158 μm within the central fovea (Fig. 1i).

## Discussion

Cyclodialysis cleft causes abnormal outflow of aqueous humour and its reduced production, resulting in serious complications, such as shallow AC, choroidal effusion or maculopathy, and consequently a reduction in visual function [6]. It should be emphasized that the IOP value does not depend on cyclodialysis cleft size [7]. Proper treatment is determined by a correct diagnosis and precise identification of the cleft. The choice of treatment depends on the ocular comorbidities and anatomic conditions. The first direct cyclopexy was reported in 1995 by Küchle and Naumann [8]. This technique facilitates direct access to a dissected ciliary body and the scleral spur [9].

Cyclopexic techniques and their modifications may be useful in the treatment of single cyclodialysis extended for 1.5 to 9.5 clock hours in pseudophakic and aphakic eyes [8, 10]. Using cyclopexy for the reconstruction of only one cyclodialysis cleft within the nasal quadrant proved to be ineffective, considering the coexistence of another dialysis elsewhere. Treatment failure could be related to the presence of a subluxated lens, vitreous prolapse into the AC as well as dialysis in a different location. It seems that each dialysis should be closed separately or another surgical procedure should be used. In the case of small cyclodialysis cleft and coexistent cataract without other ocular abnormalities, an effective technique, reported in the subject literature, seems to be phacoemulsification accompanied by implantation of a single-piece IOL with large haptics (polymethylmethacrylate, PMMA, Type 01; Alcon Laboratories, Inc.) into the capsular bag in order to close the cyclodialysis by applying a directional force on the ciliary body and pressing it to the sclera [2]. In patients with cataract and inextensive cyclodialysis, the circular surgical cyclopexy may be more difficult and may pose a considerable risk of damage to the vascular structures and intense bleeding from the ciliary body [11]. Therefore, large clefts, extending even to 360 degrees, may be repaired after phacoemulsification by inserting a large-diameter 13-mm CTR with 2-point scleral suture fixation, 1 mm posterior to the surgical limbus, with 10.0 polypropylene suture through the ciliary body and into the ciliary sulcus [2, 9].

A successful technique used for the repair of cyclodialysis cleft was implantation of a tension ring into the ciliary sulcus [11, 12]. This technique involves a 3-mm long keratotomy in the superior quadrant. Following a standard phacoemulsification, the CTR is placed in the ciliary sulcus and fixed to the sclera by means of interrupted 10.0 sutures. The sutures are placed at the largest cleft. Using this type of technique seems to be very safe, as 360-degree suturing around the perimeter involves a risk of bleeding from highly vascularized ciliary body structures. Another method is the implantation of a single-piece artificial posterior chamber lens into the ciliary sulcus [2]. In this technique, the implanted lens is rotated so that the center of one of the haptics is under the cleft. This technique was reported in the literature as suitable for both phakic and aphakic eyes, after earlier phacoemulsification. The described procedure, supported by the process of scar formation, results in a definite closure of small clefts [2]. It seems, however, that the delineated surgical techniques are effective in a situation when there are no other abnormalities in the anterior segment of the eye, such as lens subluxation, vitreous body prolapse, or pupil sphincter rupture. Hoerauf *et al*. described a patient with traumatic cyclodialysis cleft aphakia and partial aniridia, who was successfully treated with vitrectomy, cryotherapy, and gas tamponade. However, in the case of patients with phakia, the procedure must be combined with lensectomy [13]. The choice of surgical technique depends on the extent of injury and type of damage. Post-traumatic cases, accompanied by damage to the other ocular structures, require extensive surgical skills, sometimes a multistep procedure and a long recovery period.

Another surgical technique, proposed by Helbig and Foerster in 1996, is a complex procedure that involves a combination of posterior vitrectomy, cataract removal with the implantation of an artificial lens, pars plana cryopexy, and gas endotamponade [14]. It was also successfully employed by other surgeons [13, 15]. Its main objective is the performance of gas endotamponade. Following phacoemulsification and aspiration, pars plana vitrectomy is performed to achieve posterior vitreous detachment. Then the vitreous is removed together with the peripheral area. In the next step, cryopexy (–85 °C for 7 to 10 seconds) is made under transpupillary observation. After implantation of an artificial posterior chamber, lens fluid–air exchange is carried out, followed by endotamponade with 20% sulfur hexafluoride (SF_6_) [11]. It seems to be an effective technique, but it should only be used in specific situations. It is a complex long-lasting procedure that requires considerable stability of the lens diaphragm. In our case, the subluxated lens could have been too unstable and this could have resulted in gas leakage into the AC. Reduced gas volume in the vitreous chamber could result in insufficient gas pressure on the ciliary body and incomplete closure of the cyclodialysis cleft. On the other hand, pupil cerclage could disturb the communication between the AC and vitreous chamber, and thus pose a threat of malignant glaucoma. The case we have described here required complex operational procedures. Our main objective was the repair of a cyclodialysis cleft with simultaneous removal of a subluxated lens and reconstruction of our patient’s damaged pupil.

## Conclusions

In case of cyclodialysis the basic purpose is always a reduction of fluid flow from the anterior chamber and hence an increase of the intraocular pressure, preventing possible occurrence of the previously described complications and consequences. Cyclopexy combined with phacoemulsification and transscleral suturing of Cionni ring and intraocular lens implantation with iris cerclage suture can be a good solution in the cases of this type.

### What was known

1. Currently, the treatment for trauma is based on many individual multiple step operating techniques.
2. There is no one surgical technique for these trauma cases which has been recognized as the most cost efficient and least traumatic to the patient.

### What this case report adds

1. Our technique presents a comprehensive treatment for post-traumatic cyclodialysis cleft with a subluxated intracapsular cataract using IOL implantation, involving the previously described separate surgical methods.
2. This is an effective method not previously documented in any literature.

## Abbreviations

AC: Anterior chamber
BCVA: Best-corrected visual acuity
CCC: Continuous curvilinear capsulorhexis
CTR: Capsular tension ring
IOL: Intraocular lens
IOP: Intraocular pressure
OCT: Optical coherence tomography
SF_6_: Sulfur hexafluoride
UBM: Ultrasound biomicroscopy
